# AhR Activation at the Air-Blood Barrier Alters Systemic microRNA Release After Inhalation of Particulate Matter Containing Environmentally Persistent Free Radicals

**DOI:** 10.1007/s12012-025-09989-z

**Published:** 2025-04-11

**Authors:** Ankit Aryal, Ashlyn C. Harmon, Alexandra Noël, Qingzhao Yu, Kurt J. Varner, Tammy R. Dugas

**Affiliations:** 1 Department of Comparative Biomedical Sciences, School of Veterinary Medicine, Louisiana State University, Baton Rouge, LA 70803 USA; 2 Biostatistics, School of Public Health, Louisiana State University Health Sciences Center, New Orleans, LA 70112 USA; 3 Department of Pharmacology and Experimental Therapeutics, Louisiana State University Health Sciences Center, New Orleans, LA 70112 USA

**Keywords:** microRNA, Environmentally persistent free radicals, Particulate matter, Air pollution, Cardiovascular health, Aryl hydrocarbon receptor, Alveolar type-II cells, Environmental toxicology, Inhalation

## Abstract

Particulate matter containing environmentally persistent free radicals (EPFRs) is formed when organic pollutants are incompletely burned and adsorb to the surface of particles containing redox-active metals. Our prior studies showed that in mice, EPFR inhalation impaired vascular relaxation in a dose- and endothelium-dependent manner. We also observed that activation of the aryl hydrocarbon receptor (AhR) in the alveolar type-II (AT-II) cells that form the air-blood interface stimulates the release of systemic factors that promote endothelial dysfunction in vessels peripheral to the lung. AhR is a recognized regulator of microRNA (miRNA) biogenesis, and miRNA control diverse signaling pathways. We thus hypothesized that systemic EPFR-induced vascular endothelial dysfunction is initiated via AhR activation in AT-II cells, resulting in a systemic release of miRNA. Using a combustion reactor, we generated EPFR of two free radical concentrations—EPFR_lo_ (10^16–17^ radicals/g particles) and EPFR (10^18–19^ radicals/g)—and exposed mice by inhalation. EFPR inhalation resulted in changes in a distinct array of miRNA in the plasma, and these miRNAs are linked to multiple systemic effects, including cardiovascular diseases and dysregulation of cellular and molecular pathways associated with cardiovascular dysfunction. We identified 17 miRNA in plasma that were altered dependent upon both AhR activation in AT-II cells and ~ 280 ug/m^3^ EPFR exposure. Using Ingenuity Pathway Analysis, we found that 5 of these miRNAs have roles in modulating endothelin-1 and endothelial nitric oxide signaling, known regulators of endothelial function. Furthermore, EPFR exposure reduced the expression of lung adherens and gap junction proteins in control mice but not AT-II-AhR deficient mice, and reductions in barrier function may facilitate miRNA release from the lungs. In summary, our findings support that miRNA may be systemic mediators promoting endothelial dysfunction mediated via EPFR-induced AhR activation at the air-blood interface.

**Graphical Abstract:**

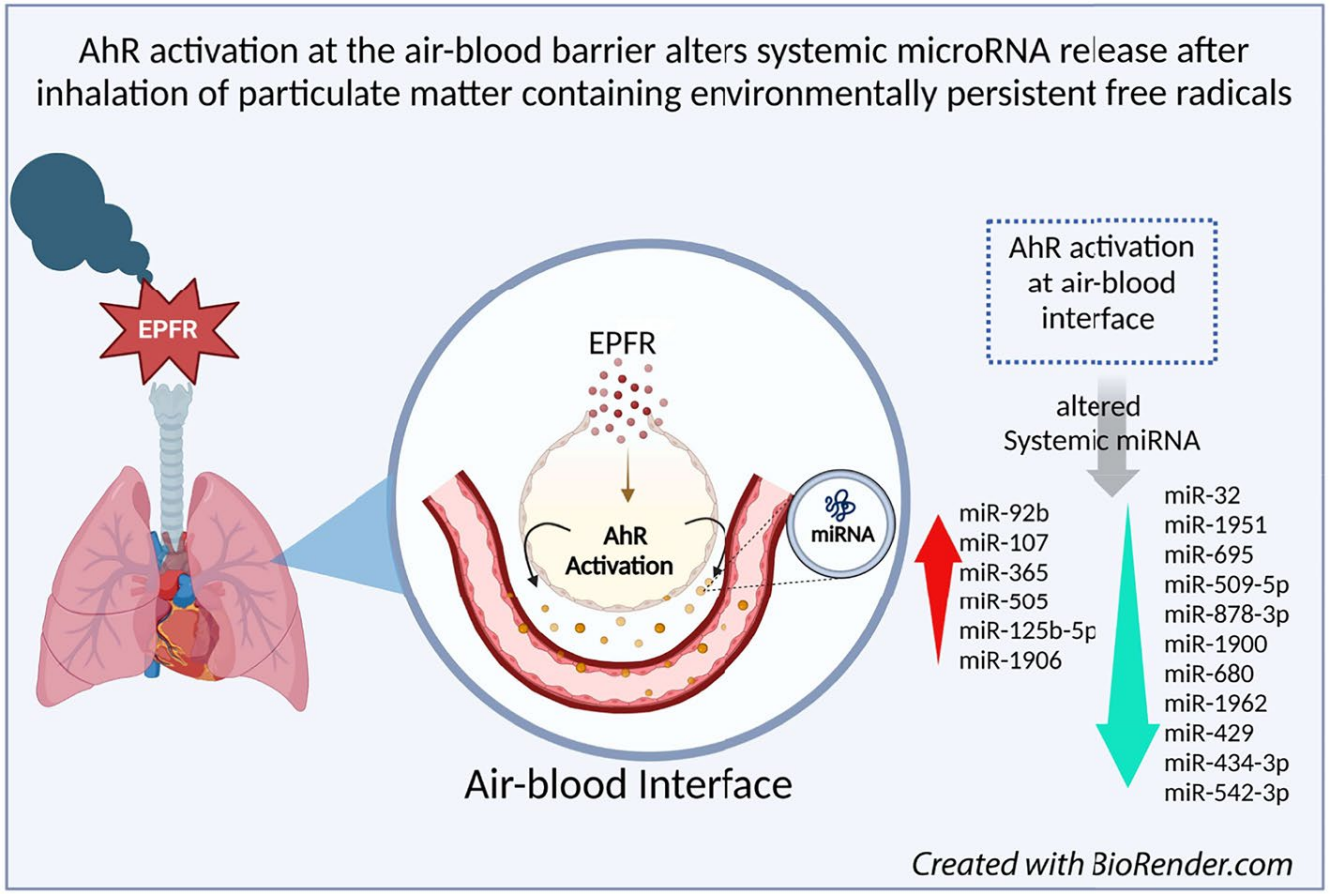

**Supplementary Information:**

The online version contains supplementary material available at 10.1007/s12012-025-09989-z.

## Introduction

Exposure to environmental stressors is a significant risk factor for disease development. In fact, the World Health Organization (WHO) identified air pollution as the most important environmental factor contributing to adverse human health and disease development. Recent data estimates that millions of premature deaths, largely due to ischemic heart disease, stroke, pneumonia, chronic obstructive pulmonary diseases, and lung cancer, occur yearly due to outdoor and household air pollution [1–3]. Epidemiological studies demonstrate that airborne particulate matter (PM) increases the risk of developing cardiovascular disease [4–6]. PM is a mixture of solid particles and liquid droplets. These particles range in size from nanometers to millimeters and form in air [7] through the incomplete combustion of organic matter, leading to a discharge of oxidized organics. These oxidation products bind to metal centers on particle surfaces and undergo electron transfer to form stabilized free radical complexes [8, 9]. These radicals persist in the air for several days to weeks and months [10, 11] and are thus referred to as environmentally persistent free radicals (EPFR). PM emissions from thermal processes like thermal remediation contain EPFR [12–14], but EPFR are also found in other combustion sources like forest fires, vehicle emissions, and cigarette and e-cigarette aerosols [15, 16]. Electron paramagnetic resonance analysis of EPFR indicates broad signals with a *g*-value at ~ 2.003, suggesting a mixture of carbon- and oxygen-centered radicals [17, 18]. The unique chemistry of EPFR enables their ability to redox cycle to continuously generate ROS. This redox cycling is maintained in biological fluids [19], and PM-containing transition metals, quinoids, and polycyclic aromatic hydrocarbons act synergistically to produce a more robust cellular ROS than that provided by the individual constituents alone [20]. However, a significant gap in knowledge remains regarding EPFR exposure and its impacts on human health.

In our previous studies, EPFR induced endothelial dysfunction and impaired lung function in a manner intricately associated with pulmonary oxidative stress and activation of the aryl hydrocarbon receptor (AhR) [21]. In mice selectively deficient in AhR expression in alveolar type-II (AT-II) cells, specialized epithelial cells residing at the air-blood interface in the lung, EPFR-induced endothelial dysfunction observed downstream of the lung was abolished [22]. These findings suggest that AhR activation in AT-II cells mediates EPFR-induced vascular endothelial dysfunction possibly through their systemic release of bioactive mediators. The AhR is a transcription factor that once activated, translocates from the cytoplasm to the nucleus where it regulates the transcription of genes with xenobiotic response elements within their promoter regions [23]. AhR activation contributes to numerous biochemical and toxic effects, such as teratogenesis, immune modulation, tumor formation, and modulation of genes involved in the metabolism of toxicants [24–28]. The AhR is also a potent regulator of miRNA biogenesis in the lungs [29].

miRNA are small non-coding RNA molecules first discovered in 1993 during an investigation of the developmental pathways of *Caenorhabditis elegans*. These miRNAs were identified as a single strand of 22 nucleotides that played a crucial role in the epigenetic regulation of gene expression [30]. miRNA primarily bind to the 3′-untranslated regions (UTRs) of target mRNA, inhibiting gene translation [31]. However, miRNA binding sites have also been identified in other regions such as 5′-UTR, coding sequences and even promoter regions of mRNA targets [32]. Such miRNA-mRNA interactions result in either a silencing of expression or enhancement of gene transcription, underscoring their versatility in gene regulation. With respect to miRNA’s role in regulating gene translation, over the last two decades, miRNA have captured widespread interest for their regulation of both normal cellular physiology and disease processes and have been extensively studied as prognostic and diagnostic biomarkers of disease. These studies have identified thousands of mature miRNA in many species such as mouse, rats, horses, and birds, including many relevant to humans [33–35].

Specific miRNA patterns are frequently used as biomarkers for diseases including cardiovascular, neurodegenerative diseases, and cancer [36–38]. miRNA are also proposed as objective measures of disease diagnosis, prognosis, and treatment options, particularly in cancer cases [39–41]. However, the relationship between exposure to PM air pollution and expression of miRNA has not been extensively studied. Emerging research suggests that miRNA targets are vital mediators of the endothelial cell cycle, nitric oxide signaling, apoptosis, and inflammation [42–44], and contribute to angiogenesis and endothelial dysfunction and activation [45, 46]. Several studies showed that circulating miRNA levels correlate with exposure to PM [47, 48]. Interestingly, the Susceptibility to Particle Health Effects, miRNA and Exosome (SPHERE) study conducted with 1630 human subjects suggested that exposure to the PM downregulates a number of miRNA, packaged within extracellular vesicles. These data further suggested that PM-induced downregulation of these miRNAs mediated increases in fibrinogen levels, thus contributing to the development of cardiovascular events [49].

Given the adverse health effects associated with air pollutant exposures, along with several methodological developments facilitating the study of miRNA, we investigated whether epigenetic markers, such as miRNA expression, contribute to EPFR-associated cardiovascular disease pathogenesis. We hypothesized that exosomal miRNA are systemic mediators that link EPFR inhalation into the deep lung with vascular dysfunction observed distal to the lung. Using mice selectively deficient in AhR within AT-II cells, we further investigated the link between EPFR-induced AhR activation in pulmonary AT-II cells and exosomal miRNA levels in the plasma.

## Materials and Methods

### Mice

All mice used in this study were purchased from Jackson Laboratories (Bar, Harbor, ME). Animals were allowed to acclimate to the facility for at least one week before experiments. For the initial screening of EPFR-induced alterations in plasma miRNA levels, we used 24 male C57BL/6J mice (*n* = 8/group). We used only male mice for this initial study because compared to females, male mice reportedly exhibit greater susceptibility to vascular dysfunction induced by ultrafine particle inhalation [50–52]. However, in subsequent studies aimed at examining the impact of AT-II-dependent AhR activation, a cohort of 20 female and 16 male mice were employed. Mice deficient in AhR in AT-II cells and their littermate controls (*n* = 5–6/group) were generated by breeding male *Ahr*^*fl/fl*^ (*Ahr*^*tm3.1Bra*^/J) mice with female Surfactant Protein C (*Sftpc*)-*Cre*-recombinase transgenic (B6.129S-*Sftpc*^*tm1(Cre/ERT2) Blh*^/J) mice, as described previously [22]. All mice were housed in HEPA-filtered air-ventilated cages in a climate-controlled facility maintained at 68–78° F and 40–60% humidity, with a normal 12 h light and 12 h dark cycle. The mice were housed on soft bedding in shoebox cages and were provided ad libitum access to regular chow diet and water. All animal experiments were conducted using protocols approved in advance by the Institutional Animal Care and Use Committee at Louisiana State University.

### Generation of Combustion-Derived EPFR

Combustion-derived EPFR particles were generated and characterized as previously described [53]. In brief, EPFR were produced from the catalytic combustion of 1-methyl naphthalene in the presence of anthracene and mono-chlorophenol. A two-stage combustion reactor was used to generate EPFR with a well-defined and -controlled free radical composition [53]. In the reactor, the first stage created iron oxide nanoparticles via high-temperature oxidation of a reverse micelle suspension Fe(III) nitrate in oleic acid and hexane, maintained at 700°C with a 60 s residence time. Those particles passed through a cooling section to ensure nucleation before reaching the next stage in the reactor. The second stage was operated under oxygen-starved combustion conditions at 975 °C with a 1 s gas-phase residence time. Combustion fuel was injected via a side port, preventing backflow into the reactor. EPFR concentration was controlled by adjusting the combustion conditions at the fuel injection point. EPFR particles used for these studies are denoted as EPFR (1e^17−18^ radical/g) and EPFR lo (1e^16−17^ radical/g), indicating the particles’ radical content and their potency to induce endothelial dysfunction after inhalation exposure [53]. Our ability to produce particles of differing free radical content allowed for a radical- (i.e., dose-) response study design. These exposures are environmentally relevant, as recent assessments of EPFR concentrations measured in Memphis, TN demonstrated that airborne PM contains 1.1e^17^–3.7e^19^ radicals/g [54].

### Inhalation Exposure System

A whole-body inhalation system consisting of an 18 L plexiglass chamber containing 16 individual steel-mesh compartments was used for these studies [21, 53]. This setup allows for the simultaneous exposure of multiple mice to the same conditions for several hours. The individual compartments also eliminated their ability to interact with (e.g., lick) one another during exposure. Mice aged 18–20 weeks were exposed simultaneously to either filtered air, EPFR lo, or EPFR for 4 h/d for 5 days. We have repeatedly shown that such exposures produce endothelial dysfunction [22, 53]. EPFR aerosols were produced using a venturi disperser and dry powder dispersion technique. An air flow rate of 7 L/min was used to create an airstream mixture to ensure the even distribution of particles throughout the chamber. The exhaust flow of 1–2 L/min was used to consistently remove the aerosolized particles from the bottom of the chamber, allowing air exchange. Within the exposure chamber, EPFR aerosol mass concentrations were continuously monitored using a TSI Dust Trak II Model 8530, and total mass concentrations were determined gravimetrically using a glass filter connected to Sensidyne Gilian BDX-II sampling pump (St Petersburg, FL). Using electron microscopy (essentially by gluing an EM grid to the glass fiber filter) and a scanning mobility particle sizer spectrometer (TSI, Inc., Shoreview, MN), we showed that aerosols produced in this manner exhibit average diameter sizes of 93 nm ± 2.58 (geometric standard deviation; GSD) and 106 nm ± 2.47 (GSD) for EPFR and EPFR lo, respectively [53].

In the initial study, C57BL/6J mice were exposed to either filtered air, EPFR lo, or EPFR, with all particle exposures maintained at a constant particle mass concentration of 280 ± 5 µg/m^3^. Follow-on studies using transgenic mice used the same particle mass concentrations, but because our screening studies in C57BL/6J mice failed to demonstrate a difference in the profile of miRNA altered by EPFR compared to EPFR lo exposures, these mice were divided into only two exposure groups—filter air or EPFR. At the end of each exposure, mice were placed back in their cages where they breathed HEPA-filtered air. After the fifth exposure, the mice were euthanized using pneumothorax under anesthesia (pentobarbital, 50 mg/kg i.p), in accordance with the American Veterinary Medical Association’s Guidelines for the Euthanasia of Animals. Blood was collected from the abdominal aorta in EDTA-coated collection tubes and plasma was obtained by centrifugation. Lungs and aorta were collected in cryovials containing RNAprotect (Qiagen), snap-frozen in liquid nitrogen, and stored at – 80 °C.

### Exosomal RNA Extraction

The Plasma/Serum RNA Purification Kit from Norgen Biotek (Canada) was used for isolating circulating and exosomal RNA from 200 µL plasma samples (*n* = 5–8/group). To eliminate any remaining cellular debris before proceeding with RNA extraction, the samples were thawed on ice and then centrifuged at 400×*g* for 10 min at 4 °C. The extraction was carried out according to the manufacturer’s guidelines. RNA concentration was determined using a NanoDrop One spectrophotometer (ThermoFisher, USA), and the quality of the RNA was determined using an Agilent 5300 fragment analyzer system (Agilent Technologies, Santa Clara, CA). Hemolyzed plasma was excluded from the study.

### miRNA Profiling

Approximately 10 ng of plasma RNA from each mouse sample (*n* = 5–8/group) was processed using the NanoString nCounter Mousev1.5 miRNA Expression Assay Kit and its manufacturer-recommended protocol. Each sample was run independently, with no pooling of samples. An nCounter Digital Analyzer was used to scan each sample for 577 miRNA. Samples were run in multiple batches and raw count data collected from independent runs were combined and processed using nSolver software (NanoString). The raw count data for each miRNA was subjected to the following processing steps: First, background subtraction was performed using the geometric mean of counts for the negative control probes (6 engineered RNA sequences). Next, miRNA counts were normalized by both positive control normalization, using the geometric mean of synthetic positive control target counts, and to apply a sample-specific correction factor to all the target probes, CodeSet Content Normalization, accomplished using the geometric mean of counts recorded for housekeeping genes (*Actb, B2 m, Gapdh, Rpl19*) present in the panel. All miRNA data and their metadata uploaded to the NCBI Gene Expression Omnibus (GEO) database under accession numbers GSE286171 and GSE286172.

### Gene Expression Analysis

Lungs tissue samples (*n* = 5–6/group) were carefully collected into cryovials containing RNA-protect solution (Qiagen, Hilden, Germany) and rapidly frozen using liquid nitrogen. Samples were then stored at – 80 °C until processed. Gene expression of tight junction proteins (tight junction protein 1; *Tjp1* (Mm01320638_m1, ThermoFisher, USA)*,* occludin domain containing 1: *Ocel1* (Mm01349279_m1, ThermoFisher, USA) were evaluated in lungs to correlate the miRNA release from the lungs into the systemic circulation, mediating EPFR-induced endothelial dysfunction. RNA extraction from tissue samples was performed using the RNeasy Mini kit (Qiagen, Hilden, Germany). Next, the concentration and purity of the extracted RNA was assessed using a Nanodrop spectrophotometer (ThermoFisher, USA) and Agilent 5300 fragment analyzer system (Agilent Technologies, Santa Clara, CA). For cDNA synthesis, 1 µg of RNA was employed, and the iScript Reverse Transcription Supermix (Bio-Rad, CA, USA) was used for this purpose. The resulting cDNA was diluted with RNase-free water. To analyze gene expression levels, 25 ng of cDNA was subjected to real-time PCR using a Biorad CFX96 Touch PCR machine (Bio-Rad, CA, USA). The iTaqMan Universal Probe Supermix (Bio-Rad, CA, USA) was utilized for PCR reactions. Real-time PCR amplification settings included an initial cycle for polymerase activation and DNA denaturation at 95 °C for 30 s, followed by 40 cycles of amplification, comprising denaturation at 95 °C for 5 s and annealing at 60 °C for 30 s. Hypoxanthine phosphoribosyl transferase (*Hprt,* Mm03024075_m1, ThermoFisher, USA) served as the internal reference gene to normalize the results obtained. Gene expression was calculated using the ∆∆Ct method, and the fold change was determined using the 2^−∆∆Ct^ method.

### miRNA Levels in AT-II Cells and Lung Microvascular Endothelial Cells

AT-II cells were isolated from three AT-II specific AhR-deficient and three wild-type mice, as described previously [22, 55]. Total RNA was extracted from isolated AT-II cells and mouse lung microvascular endothelial cells (mLMVECs; Creative Biolabs, Shirley, NY) using the RNAqueous-Micro Total RNA isolation (ThermoFisher). The quantity and quality of the isolated RNA were determined using a Nanodrop One (Nanodrop Technologies, Wilmington, Delaware) and Agilent 5300 Fragment Analyzer (Agilent Technologies, Santa Clara, California), respectively. RNA isolated from samples was then normalized to 175 ng/uL RNA, and cDNA was made using the Mir-X miRNA First-Strand Synthesis (TAKARA). Quantitation was performed in duplicate for miR-1906, miR-542-3p, and the spiked-in housekeeping gene, U6, using TB Green Advantage qPCR Premix Kit and 50 ng of cDNA per well. AT-II expression of miR-1906 and miR-542-3p was used as the baseline for comparing ATII to mLMVEC. Data were analyzed using the threshold cycle values for each well and the 2^–(Δ ΔcT)^ method to establish fold changes.

### Statistical Analysis

Data were analyzed using one-way or mixed effects ANOVA with Tukey’s post hoc tests to determine significance among groups. Statistical analyses were conducted using GraphPad Prism 9.0 (La Jolla, CA) or R (version 4.3.1, Austria). All data were expressed as means ± standard error of the mean (SEM); in all cases, *p*-value < 0.05 was considered statistically significant. Bonferroni adjustment was used to control the familywise type I error rate for multiple comparisons.

### Ingenuity Pathway Analysis (IPA)

To gain insights into the biological networks and pathways possibly impacted by EPFR-induced alterations in miRNA expression, differentially expressed miRNA were analyzed using IPA software. IPA Core analysis was used to understand the interaction between systemically differentiated miRNA in annotated diseases as well as the molecular and cellular functions associated with them. To identify the target genes of differentially expressed miRNA, we used the IPA microRNA target filter that incorporates a comprehensive dataset including experimentally validated targets from TarBase and miRecords sources, predicted targets from TargetScan, and miRNA-mRNA interactions manually curated from peer-reviewed literature [56–59]. IPA considered the seed sequence of the miRNA to filter target mRNA that are predicted to play an important role in endothelin-1 (ET-1) and endothelial nitric oxide signaling (eNOS). The seed sequence is a conserved heptametrical nucleotide sequence typically located between positions 2–8 from the miRNA’s 5′- end and is a crucial element required for miRNA binding to its target mRNA.

## Results

### EPFR Exposure Induces a Unique and Specific Pattern of Circulating miRNA

**Fig. 1:**
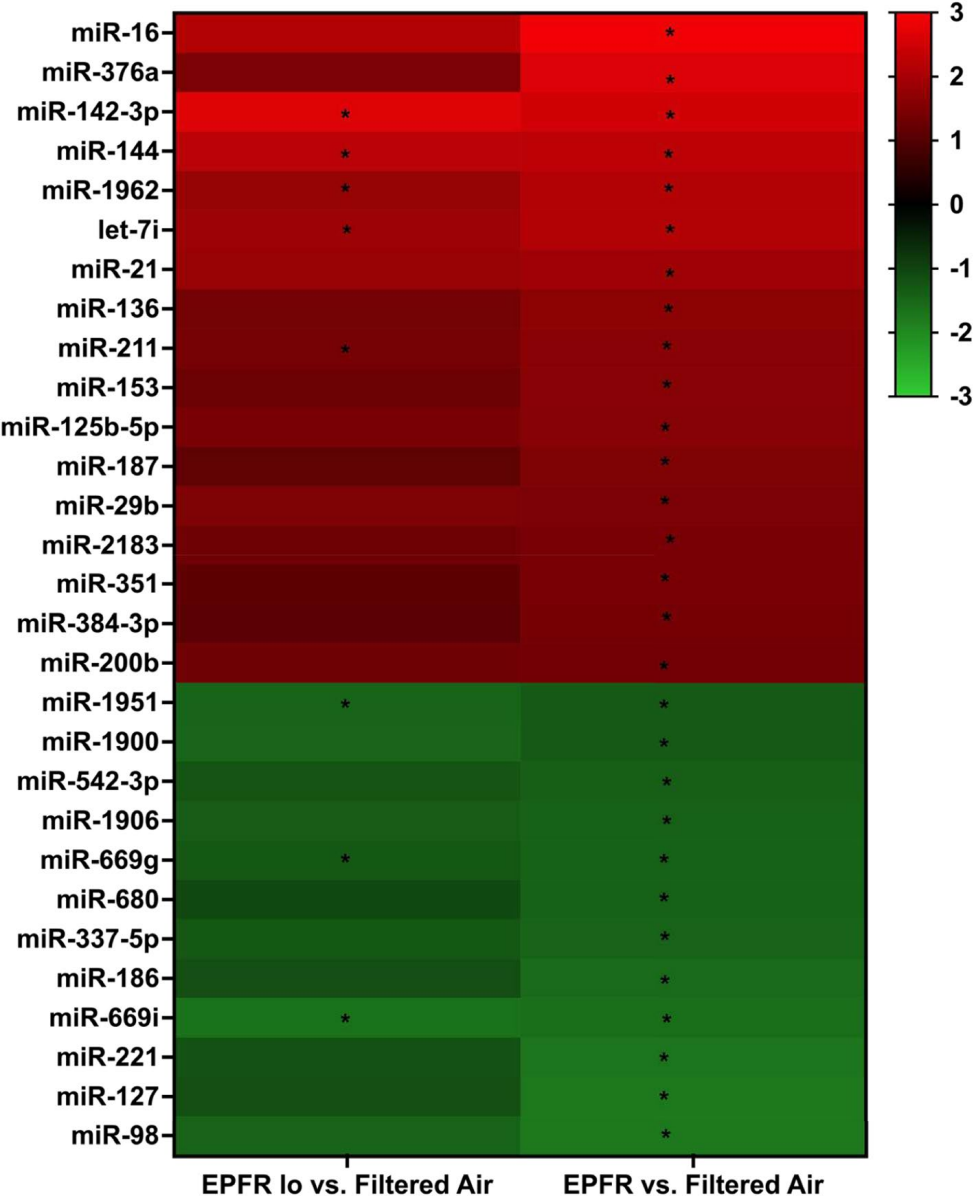
EPFR exposure alters levels of exosomal miRNA in plasma. Exosomal miRNA was extracted from plasma of male C57BL/6J mice (*n* = 8/group) exposed to EPFR for 5 days. miRNA levels are expressed as fold changes. Data were analyzed using one-way ANOVA with Tukey’s post hoc tests. The heatmap shown was generated from miRNA whose levels were significantly altered in mice exposed to EPFR lo (left) or EPFR (right). *p < 0.05

To discern changes in circulating miRNA profiles induced by EPFR, we examined miRNA transcripts in plasma of C57BL/6J mice subjected to inhalation of EPFR, EPFR lo, and filtered air using the NanoString nCounter miRNA profiler. Among the 577 distinct miRNA identified in plasma, levels of 29 miRNA were significantly altered by EPFR exposure (Fig. 1), with 17 significantly increased and 12 significantly decreased compared to filtered air exposure. For mice exposed to EPFR lo, plasma levels of only 8 miRNA were significantly altered compared to filtered air exposed mice. Additionally, for nearly all miRNA significantly altered in the EPFR treatment, levels measured in the EPFR lo exposure group trended in a similar manner, with *p* values approaching significance (i.e., with *p* = 0.059–0.09). In other words, miRNA whose plasma levels were significantly increased in the EPFR exposure group also trended higher for EPFR lo exposed mice, and those whose levels that were significantly decreased for EPFR treatment trended lower for mice exposed to EPFR lo. Moreover, we identified no miRNA whose levels were altered in EPFR lo but not EPFR exposed mice.

### IPA Suggests a Potential Role for miRNA as Systemic Mediators for the Health Effects of EPFR Inhalation

**Fig. 2:**
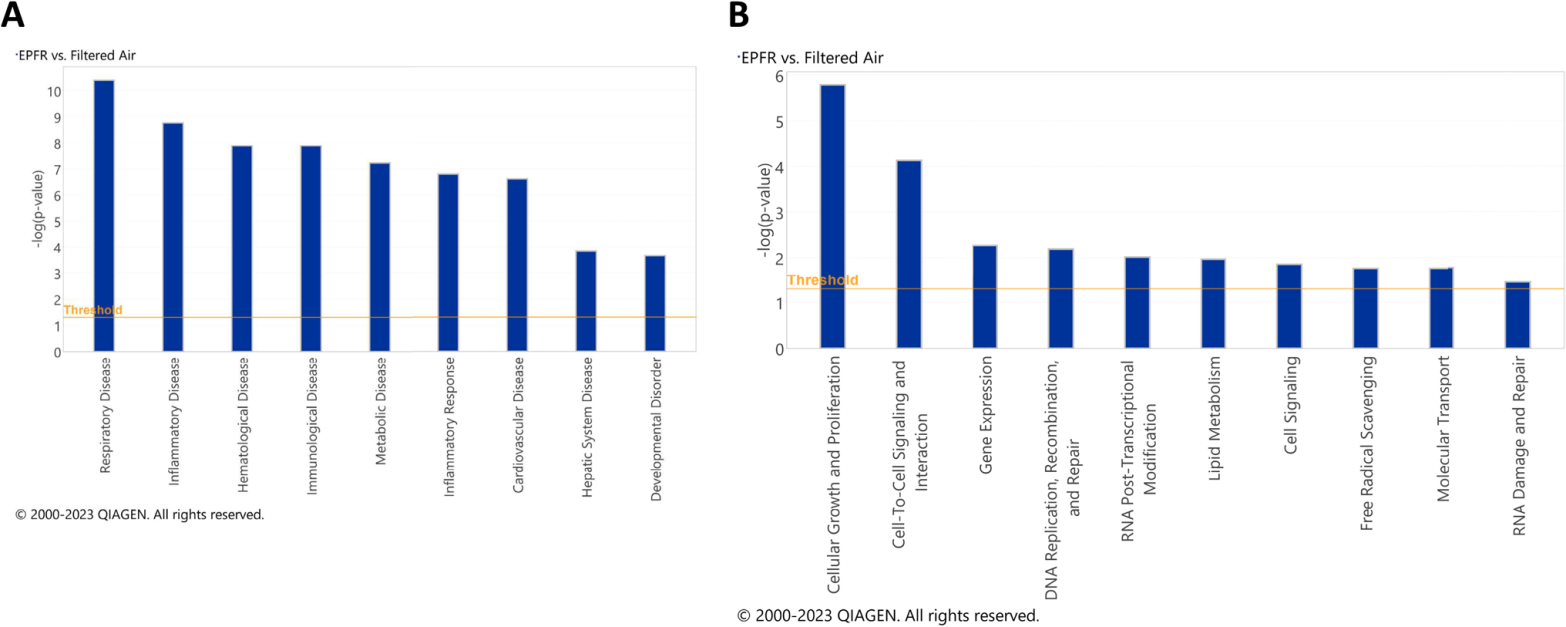
Ingenuity pathway analysis (IPA) reveals systemic health effects **A** as well as molecular and cellular functions **B** potentially impacted by EPFR-induced alterations in miRNA levels. The graph illustrates category scores, where the threshold represents the minimum significance level (measured as –log (*p*-value) derived from Fisher’s exact test) set at 1.25

EPFR-associated alterations in plasma miRNA were further interrogated using IPA Core Analysis to correlate miRNA changes with potential systemic diseases and disorders, and molecular and cellular functions. The most prominent disease associations identified were respiratory, hematological, immunological, metabolic, cardiovascular, and hepatic system diseases, as well as inflammatory responses and developmental disorders (Fig. 2A). EPFR-induced alterations in systemic miRNA profiles were also linked with altered molecular and cellular pathways. The most strongly correlated pathways were related to cellular growth and proliferation, cell-to-cell signaling and interaction, gene expression, DNA replication, recombination and repair, RNA post-transcriptional modification, lipid metabolism, cell signaling, free radical scavenging, molecular transport and RNA damage and repair (Fig. 2B).

### AhR Activation in AT-II Cells Mediates EPFR-Induced Alterations in Systemic miRNA

**Fig. 3:**
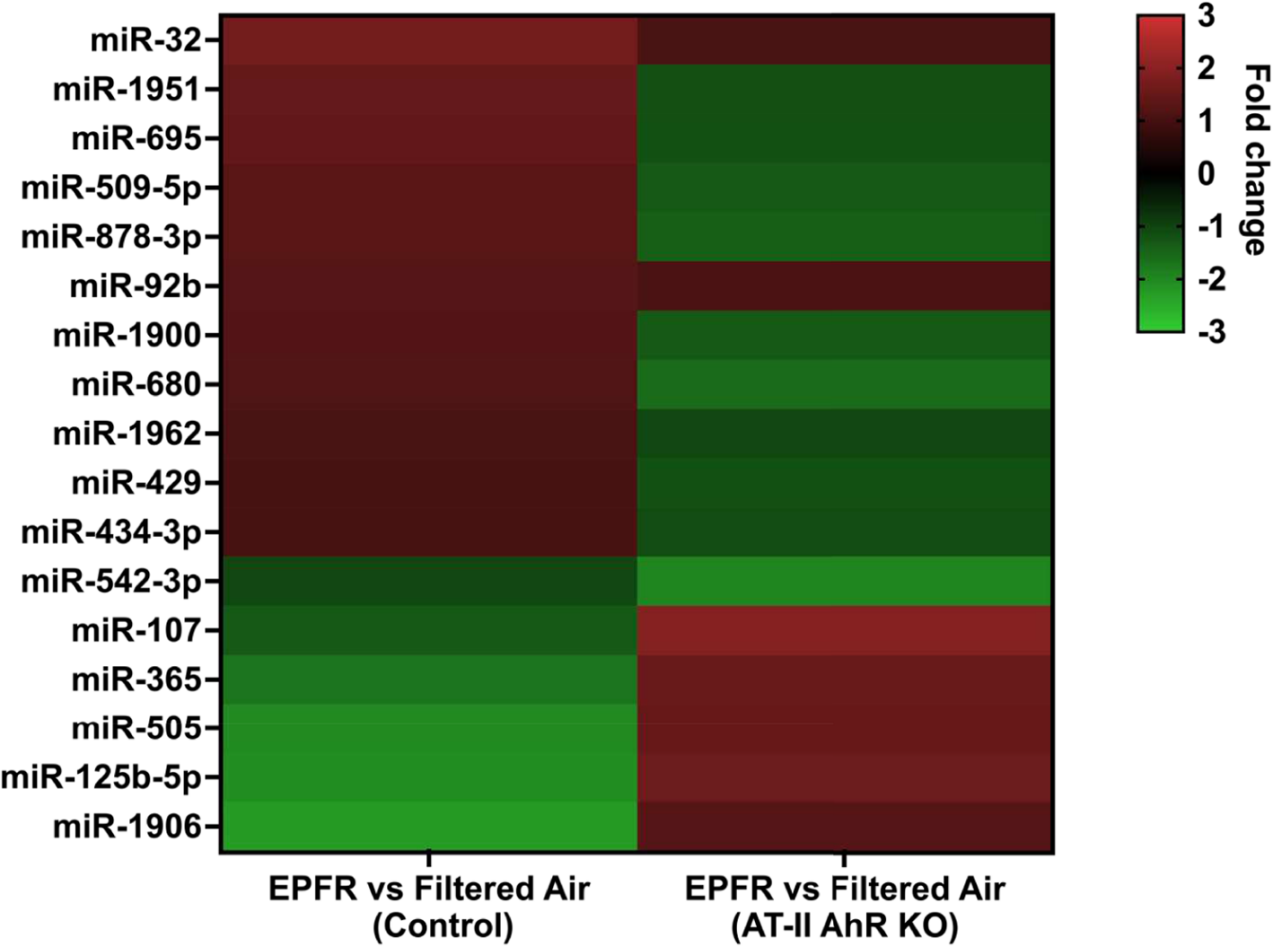
EPFR-mediated AhR activation produces a distinct miRNA signature. Male (*n* = 16) and female (*n* = 20) mice deficient in AhR in alveolar type-II cells (AT-II AhR KO; *n* = 5–6/group) and littermate control mice (Control; *n* = 5–6/group) were exposed to EPFR versus filtered air for 5 consecutive days. A heat map was generated for miRNA significantly altered by EPFR exposure in each strain, assessed as a significant interaction detected using two-way ANOVA. *p < 0.05

To specifically identify the subset of circulating miRNA whose level of expression was changed by EPFR-associated AhR activation in AT-II cells, we profiled the miRNA in plasma collected from control and AT-II cell-specific AhR knockout mice exposed to EPFR. Among 577 miR, we observed a significant reduction in 5 miRNA (miR-181a, miR-2132, miR-1939, miR-2137, and miR-18b) in AT-II cell-specific AhR KO compared to control mice exposed to filtered air only (data not shown). However, EPFR exposure increased the expression of 6 miRNA (miR-92b, miR-107, miR-365, miR-505, miR-125b-5p, and miR-1906) and decreased the expression of 11 miRNA (miR-32, miR-1951, miR-695, miR-509-5p, miR-878-3p, miR-1900, miR-680, miR-1962, miR-429, miR-434-3p, and miR-542-3p) in an AT-II cell-specific and AhR-dependent manner (Fig. 3).

### AhR-Dependent miRNA may Promote EPFR-Induced Endothelial Dysfunction

**Fig. 4:**
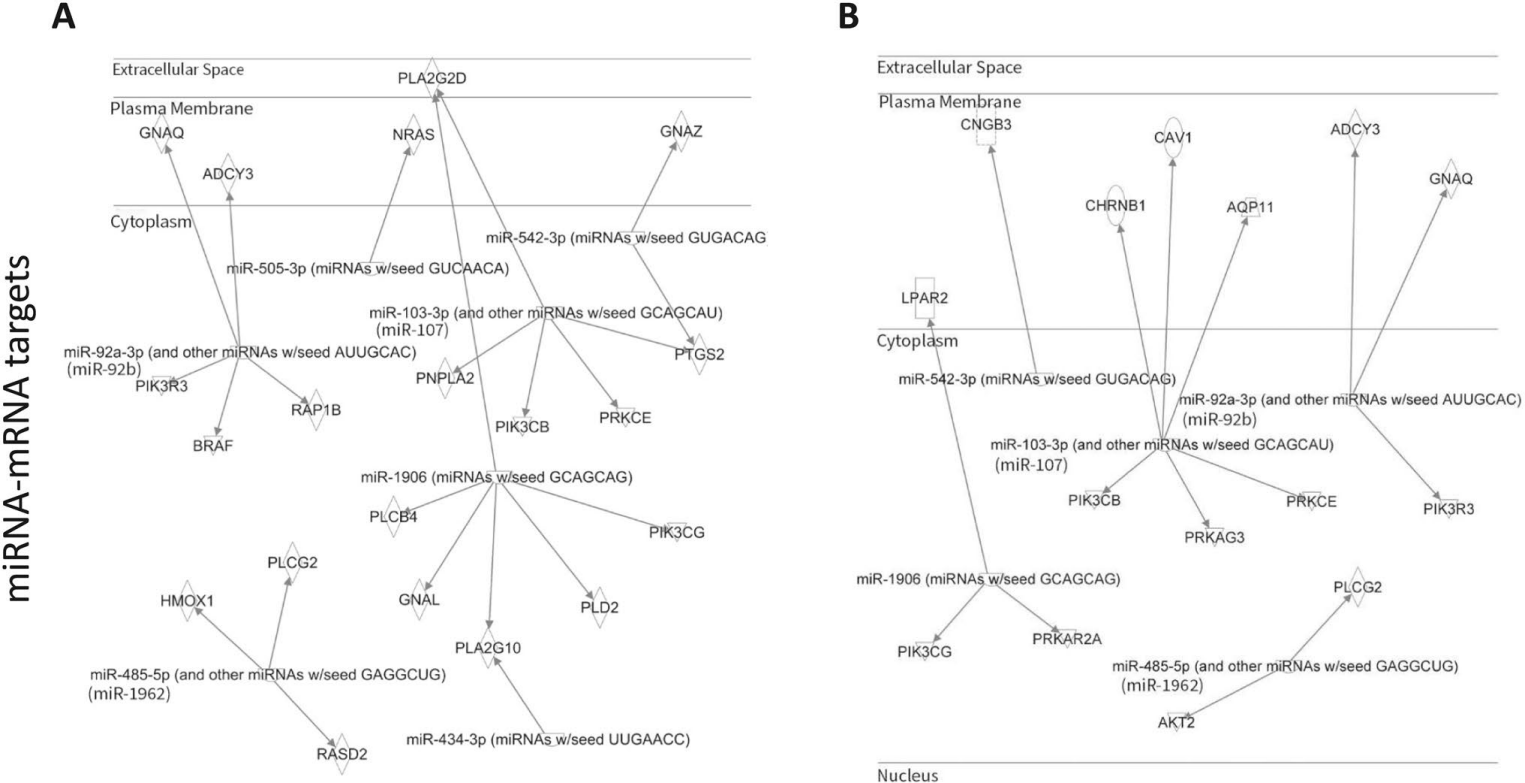
AhR-responsive miRNA is linked to target genes known to be important in regulating endothelial function. miRNA whose levels were altered in an EPFR- and AhR-dependent manner were subjected to an IPA microRNA target filter to illuminate potential mRNA targets. Pathway analysis suggested potential gene targets associated with endothelin-1 signaling (**A**) and endothelial nitric oxide signaling pathways (**B**). GNAQ guanine nucleotide binding protein alpha q subunit, GNAZ guanine nucleotide binding protein alpha z subunit, ADCY3 adenylate cyclase 3, NRAS neuroblastoma Ras oncogene, PLA2G2D phospholipase A2 group IID, PIK3R3 phosphoinositide-3-kinase regulatory subunit 3, BRAF B-Raf transforming gene, RAP1B RAS related protein 1b, PNPLA2 patatin-like phospholipase domain containing 2, PIK3CB phosphatidylinositol-4,5-bisphosphate 3-kinase catalytic subunit beta, PIK3CG phosphatidylinositol-4,5-bisphosphate 3-kinase catalytic subunit gamma, PRKCE protein kinase C epsilon, PTGS2 prostaglandin-endoperoxide synthase 2, HMOX1 heme oxygenase 1, PLCG2 phospholipase C gamma 2, RASD2 RASD family member 2, PLA2G10 phospholipase A2 group X, GNAL guanine nucleotide binding protein alpha stimulating olfactory type, PLCB4 phospholipase C beta 4, PLD2 phospholipase D2, CNGB3 cyclic nucleotide gated channel beta 3, CHRNB1 cholinergic receptor nicotinic beta 1 subunit, CAV1 caveolin 1, AQP11 aquaporin 11, LPAR2 lysophosphatidic acid receptor 2, PRKAG3 protein kinase AMP-activated gamma 3 non-catalytic subunit, PRKAR2A protein kinase cAMP-dependent regulatory type II alpha

Our prior research employing AT-II cell-specific AhR knockout mice demonstrated that EPFR inhalation promotes endothelial dysfunction in arteries peripheral to the lungs by activating AhR at the air-blood interface [22]. This study also suggested the mechanism involves an alteration in endothelin-1 (ET-1) and nitric oxide (NO) signaling in endothelial cells via paracrine mechanisms, possibly mediated via bioactive systemic mediators. IPA was conducted on those miRNAs whose expression was found to be increased or decreased in an EPFR- and AhR-dependent manner. Using IPA microRNA target filter analysis, we sought to identify the potential mRNA targets associated with these miRNAs, particularly those that may play roles in dysregulating endothelin-1 and endothelial nitric oxide signaling. To predict potential mRNA targets, IPA considered the seed sequence (2–8 nucleotides) of the altered miRNA and examined the known physiologic effects of miRNA of the same seed sequence. For example, miR-103-3p shares a similar seed sequence with miR-107, miR-92a-3p with miR-92b, and miR-485-5p with miR-1962. The IPA analysis suggested that miR-92b (shown as miR-92a-3p), miR-505-3p, miR-542-3p, miR-107 (shown as miR-103-3p), miR-1906, miR-192 (shown as miR-485-3p), and miR-434-3p have potential mRNA targets that regulate endothelin-1 signaling (Fig. 4A). Moreover, miR-92b (shown as miR-92a-3p), miR-107 (shown as miR-103-3p), miR-1906, miR-542-3p, and miR-1962 (shown as miR-485-3p) may control endothelial nitric oxide signaling by targeting genes involved in its regulation (Fig. 4B).

### EPFR Induces a Reduction in Lung Adherens and Gap Junctions

**Fig. 5:**
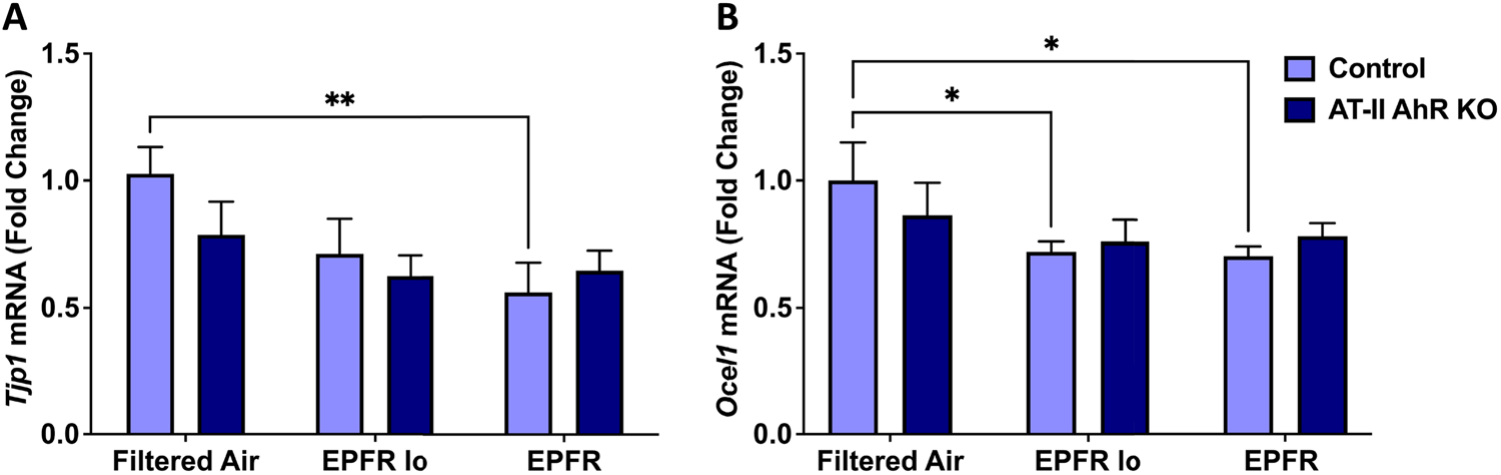
EPFR reduced the expression of genes associated with lung adherens and tight junction proteins. AT-II cell-specific AhR knockout and littermate control male (*n* = 16) and female (*n* = 20) mice (*n* = 5–6/group) were exposed to EPFR, EPFR lo, or filtered air for 5 consecutive days. mRNA levels for *Tjp1* (**A**) and *Ocel1* (**B**) were normalized to the *Hprt* gene. Data represent means ± SEM. *p < 0.05, **p < 0.01

To determine the role of lung barrier function in facilitating downstream EPFR-induced vascular dysfunction via the release of systemic mediators, we determined mRNA levels for genes involved in regulating tight junctions in lung tissues isolated from AT-II cell-specific AhR knockout and their littermate control mice (*n* = 5–6/group) subjected to EPFR inhalation. EPFR exposure significantly reduced levels of *Tjp1* and *Ocel1* mRNA levels in the lungs of control mice (Fig. 5A, B) treated with EPFR; however, *Tjp1* and *Ocel1* expression were unchanged in the lungs of AT-II cell-specific AhR knockout mice (Fig. 5A, B).

## Discussion

The respiratory epithelium, where ultrafine particles are deposited, acts as the primary gatekeeper for the AhR [60]. Our previous studies identified that AhR activation in alveolar epithelial cells at the air-blood interface regulates endothelial dysfunction observed distal to the lungs, potentially through the release of systemic mediators [22]. Furthermore, significant AhR activation in the alveolar epithelial cells was observed, particularly in AT-II cells [53], of mice exposed to EPFR. Alveolar epithelial cells are vital for gas exchange in the lung, and these cells, together with microvascular endothelial cells, create a critical interface between the air and blood compartments [61]. After exposure to harmful agents, AT-I cells are lost, but AT-II cells transform into AT-I cells to restore the air-blood barrier and secrete bioactive molecules for maintaining homeostasis [62, 63]. Due to their secretory function, our first working hypothesis was that EPFR-mediated AhR activation in AT-II cells could contribute to vascular endothelial dysfunction distal to the lung, perhaps through its regulation of miRNA biogenesis [64]. This study aimed to investigate exosomal miRNA as systemic mediators linking AhR activation at the air-blood interface with EPFR-induced endothelial dysfunction in arteries peripheral to the lung.

miRNA are short, non-coding RNA that influence gene expression primarily by targeting the 3′ untranslated region of mRNA molecules [65]. While the functional roles of miRNA are well-studied in the context of cardiovascular function [66], their contributions to the development and exacerbation of EPFR-induced endothelial dysfunction remain elusive. We identified dose-dependent alteration in 29 plasma miRNA levels, with 17 significantly increased and 12 significantly decreased after EPFR inhalation. Additionally, 8 miRNA (5 increased, 3 decreased) were significantly altered exclusively after EPFR lo exposure, with these trends mirroring and completely overlapping with those observed in the EPFR group. Furthermore, IPA suggested that these miRNAs play roles in promoting systemic health effects. Furthermore, sustained dysregulation of these miRNAs has the potential to culminate in respiratory, inflammatory, hematological, immunological, metabolic, inflammatory, cardiovascular, hepatic system, and developmental disorders. IPA Disease and Disorder Pathways Analysis also suggested that these miRNAs may be systemic mediators contributing to EPFR-induced endothelial dysfunction, which could culminate in EPFR-mediated cardiovascular disease. Individual miRNA interact with multiple mRNA targets, frequently encompassing numerous constituents of intricate intracellular networks [67]. IPA predicted that EPFR-induced miRNA can affect cellular growth and proliferation, cell-to-cell signaling and interaction, gene expression, DNA replication, recombination and repair, RNA post-transcriptional modification, lipid metabolism, cell signaling, free radical scavenging, molecular transport, and RNA damage and repair. Dysregulation of these biological mechanisms and their associated signaling pathways could culminate in systemic effects, including cardiovascular dysfunction.

Endothelial dysfunction serves as a pivotal indicator and a critical first step in the initiation of cardiovascular diseases [68]. Assessing endothelial function often involves gauging plasma concentrations of ET-1, a peptide associated with vasoconstriction, and NO, a bioproduct linked with vasorelaxation [69], as an imbalance between these two molecules generally suggests endothelial dysfunction [70]. In our previous study, EPFR-induced alterations in endothelin-1 and NO plasma levels were mediated by AhR activation in AT-II cells, potentially through the systemic mediators [22]. We observed radical dose-dependent changes in systemic miRNA upon EPFR exposure, with a lower magnitude, and no unique miRNA changes detected with EPFR lo exposure compared to EPFR. Hence, to further investigate miRNA changes associated with EPFR exposure and their potential regulation by AhR activation at the air-blood interface, we exposed AT-II cell-specific AhR knockout and their littermate control mice to EPFR exposure only. Under baseline conditions, i.e., in mice exposed to filtered air, we observed a significant reduction in miR-181a, miR-2132, miR-1939, miR-2137, and miR-18b in AT-II cell-specific AhR KO mice compared to control mice. However, upon exposure to EPFR, we identified 17 miRNAs regulated through ATII-AhR- and EPFR-dependent mechanisms. Specifically, AhR in AT-II cells positively regulated miR-92b, miR-107, miR-365, miR-505, miR-125b-5p, and miR-1906, while negatively regulating miR-32, miR-1951, miR-695, miR-509-5p, miR-878-3p, miR-1900, miR-680, miR-1962, miR-429, miR-434-3p, and miR-542-3p upon EPFR exposure. The distinct miRNA expression patterns observed under basal deletion conditions and following EPFR exposure highlight the context-dependent regulatory role of AhR. These findings further suggest that AhR activation induced by EPFR governs specific transcriptional pathways for miRNA regulation, distinct from its baseline regulatory functions.

The AhR operates as a ligand-activated transcription factor that governs the toxic consequences of numerous environmental pollutants [71]. Intriguingly, in prior studies in our laboratory, AhR activation hinged on EPFR-induced reactive oxygen species production rather than the EPFR’s chemical attributes [72]. AhR is a notable influencer of inflammation, cellular differentiation, and carcinogenesis [73]. AhR activation is also linked to epigenetic alterations, including the regulation of miRNA [74, 75]. Studies indicate that miRNA-directed signaling pathways are pivotal for endothelial cell functions encompassing cell cycle regulation, nitric oxide signaling, apoptosis, and inflammation, culminating in angiogenesis, endothelial proliferation, and endothelial dysfunction [76–78]. Furthermore, IPA miRNA-mRNA target analysis revealed that AhR-dependent alterations in our observed miRNA levels have potential mRNA targets playing a role in ET-1 and NO signaling. IPA ET-1 Pathway analysis revealed that AT-II AhR-dependent alterations in miRNA including miR-92b, miR-505-3p, miR-542-3p, miR-107, miR-1906, miR-192, and miR-434-3p can influence the mRNA target required for proper ET-1 signaling pathway. On the other hand, we found that miR-92b, miR-107, miR-1906, miR-542-3p, and miR-1962 were altered in an AT-II cell-specific AhR-dependent fashion following EPFR exposure and IPA eNOS Pathway Analysis revealed that these miRNAs have the potential to modify the mRNA gene targets regulating proper eNOS pathway.

It was interesting to note that the miRNA altered in the plasma of C57BL6/J and of control AhR floxed mice exposed to EPFRs were not identical. We posit that these differences may be because the two strains of mice express differing *Ahr* alleles. C57BL/6 mice exhibit the *Ahr*^*b*^ allele, while the AhR floxed mice we used for this study express the *Ahr*^*d*^ allele [79]. This allelic variation may introduce variations in miRNA expression, as the biogenesis of these molecules is intricately linked to AhR activation [22, 79, 80]. However, comparing the profile of miRNA altered in an EPFR and AT-II cell-specific AhR-dependent manner to our C57BL/6J miRNA dataset, we noted similar results for miR-107, miR-1906, miR-505-3p, miR-542-3p, and miR-92b. Notably, IPA suggested their interaction with genes associated with ET-1 and NO signaling pathways. This may imply an essential role for these miRNAs in mediating EPFR-induced endothelial dysfunction. In support of this assertion, increased miR-1906 and reduced miR-542-3p levels are implicated in promoting vascular function in cardiovascular diseases such as stroke, ischemic injury, and myocardial infarction [81–85], and both of these miRNAs were altered in an AT-II-AhR-dependent manner. miRNA biogenesis is a complex process; however, identifying their major cellular sources could significantly aid in developing targeted strategies to modulate their biogenesis and function.

To confirm that these miRNAs were expressed in relevant pulmonary cell types, we analyzed the expression of miR-1906 and miR-542-3p in AT-II cells we isolated from AT-II cell-specific AhR-deficient and control mice and compared their levels to that measured in mouse lung microvascular endothelial cells (mLMVEC) we obtained from a commercial source. miR-1906 and 542-3p were expressed in both cell types, but not surprisingly, their levels were higher in the freshly isolated AT-II cells (Supplemental Fig. 1A and B). We acknowledge the limitation of comparing freshly isolated AT-II cells with commercially sourced microvascular endothelial cells, so recognizing that the magnitude of expression we observed may be an artifact of passage number. Intriguingly, miR-542-3p expression was significantly reduced in AT-II cells deficient in *Ahr* (Supplemental Fig. 1B). These data confirm that both pulmonary cell types express miR-1906 and miR-542-3p and that levels of miR-542-3p may be regulated by *Ahr*. This suggests a potential role for AT-II cell-specific *Ahr* expression in modulating miRNA profiles in plasma following EPFR inhalation. Additional studies are critical for understanding the link between AhR activation in AT-II cells in pulmonary miRNA biogenesis and systemic release and the specific roles of these miRNAs in EPFR-induced endothelial dysfunction observed peripheral to the lung.

Our second working hypothesis was that an AhR-dependent disruption in barrier function may facilitate miRNA transit into the circulation. At the air-blood interface, AT-II cells serve as an important barrier, but lung microvascular endothelial cells also have roles in restricting entry into the circulation. We and our colleagues have shown that EPFR trigger a reduction in lung barrier function [86]. We thus examined the expression of genes associated with adherens junction proteins in the lungs. Our observation of reduced mRNA levels of *Tjp1* and *Ocel1* in the lungs of control but not AT-II AhR-deficient mice exposed to EPFR suggests that barrier function may be altered in an alveolar AhR-dependent manner. Studies support that AhR signaling regulates tight junction proteins at a post-transcriptional level via miRNA modulation, in turn regulating tight junction protein levels and influencing the epithelial/endothelial barrier integrity at the air-blood interface [87–93]. Intriguingly, AhR-dependent regulation of certain miRNA may itself contribute to decreasing barrier function to promote the entry of other miRNA. For example, miRNA play a crucial role in modulating mitogen-activated protein kinase (MAPK) signaling pathways essential for maintaining barrier integrity, [94, 95], and miR-200b and miR-125b have been identified as key regulators of MAPK signaling [96–98]. In this study, EPFR inhalation altered the plasma levels of both miR-200b and miR-125b (Fig. 1), and miR-125b levels appear regulated by AT-II cell-specific expression of *Ahr* (Fig. 3). Thus, while our data highlights that AhR in AT-II cells may regulate the biogenesis/secretion of systemic mediators, its activation may also regulate their release into the systemic circulation through its modulation of the air-blood barrier.

A limitation of our study is the relatively small sample size in AhR deficient mice of each sex. This limitation restricts our ability to explicitly delineate sex-specific AhR-dependent miRNA responses, thereby curtailing our capacity to interrogate sex-specific AhR-dependent miRNA expression. Consequently, caution must be exercised when extrapolating sex-specific miRNA responses mediated by AhR activation. We also acknowledge that our study design limited out ability to establish the causality of miRNA release systemically in EPFR-induced endothelial dysfunction. Hence, future investigations will delve into elucidating the mechanistic role of the identified miRNA in vascular physiological processes associated with EPFR-mediated endothelial dysfunction. Studies will likely also be required to elucidate the role of adherens junction integrity in the lungs in mediating miRNA release into the systemic circulation, providing a more comprehensive understanding of their systemic vascular effects. Also unclear is the persistence of the miRNA response after EPFR exposures and whether these responses are contingent upon sex or pre-existing disease.

## Conclusion

In summary, the studies outlined here highlight a potential role for systemic exosomal miRNA in mediating endothelial dysfunction induced by EPFR inhalation. We observed alterations in 29 miRNA in the plasma of mice exposed to EPFR by inhalation and we identified 17 that were altered through an EPFR-induced AhR activation in AT-II cells. Using pathway analysis, we found that 5 of these AhR-dependent miRNA appear to play roles in modulating the endothelin-1 and endothelial nitric oxide signaling pathways (Table 1), two pathways important in EPFR-induced endothelial dysfunction [22]. Our findings thus suggest that EPFR-induced AhR activation in AT-II cells at the air-blood interface can lead to vascular endothelial dysfunction beyond the lungs, potentially mediated by exosomal miRNA.

**Table 1:**
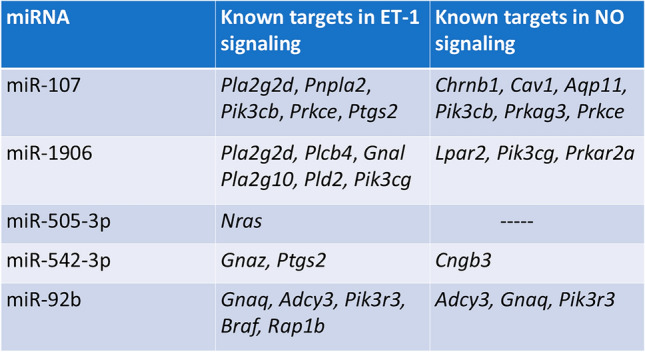
Ingenuity Pathway Analysis reveals that miRNA exhibiting comparable profiling in C57BL/6J and transgenic mice likely target genes involved in endothelin-1 (ET-1) and nitric oxide (NO) signaling

*Pla2g2d* phospholipase A2 group IID, *Pnpla2* patatin-like phospholipase domain containing 2, *Pik3cb* phosphatidylinositol-4,5-bisphosphate 3-kinase catalytic subunit beta, *Prkce* protein kinase C epsilon, *Ptgs2* prostaglandin-endoperoxide synthase 2, *Plcb4* phospholipase C beta 4, *Gnal* guanine nucleotide binding protein alpha stimulating olfactory type, *Pla2g10* phospholipase A2 group X, *Pld2* phospholipase D2, *Pik3cg* phosphatidylinositol-4,5-bisphosphate 3-kinase catalytic subunit gamma, *Nras* neuroblastoma Ras oncogene, *Gnaz* guanine nucleotide binding protein alpha z subunit, *Gnaq* guanine nucleotide binding protein alpha q subunit, *Adcy3* adenylate cyclase 3, *Pik3r3* phosphoinositide-3-kinase regulatory subunit 3, *Braf* B-Raf transforming gene, *Rap1b* RAS related protein 1b, *Chrnb1* cholinergic receptor nicotinic beta 1 subunit, *Cav1* caveolin 1, *Aqp11* aquaporin 11, *Prkag3* protein kinase AMP-activated gamma 3 non-catalytic subunit, *Lpar2* lysophosphatidic acid receptor 2, *Prkar2a* protein kinase cAMP-dependent regulatory type II alpha, *Cngb3* cyclic nucleotide gated channel beta 3

## Supplementary Information

Below is the link to the electronic supplementary material.

Supplementary file1 (TIF 149 kb)

## Data Availability

The miRNA data from this study can be accessed from the NCBI Gene Expression Omnibus (GEO) database under Accession Numbers GSE286171 and GSE286172.
