# Supplementary figures and images for: AhR Activation at the Air-Blood Barrier Alters Systemic microRNA Release After Inhalation of Particulate Matter Containing Environmentally Persistent Free Radicals
Source: Cardiovasc Toxicol. 2025 Apr 11;25(5):651–65. doi: 10.1007/s12012-025-09989-z (PMC12018632; doi:10.1007/s12012-025-09989-z)

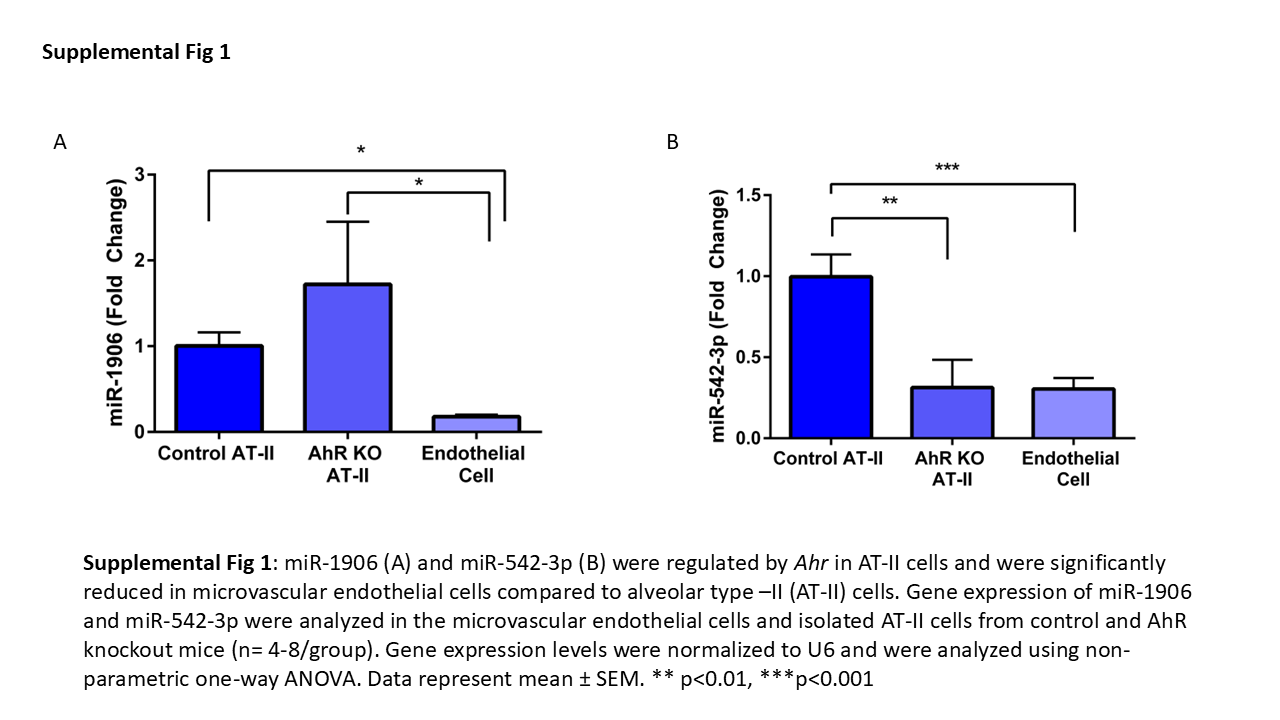

Supplement: Supplementary file 1 — Supplementary file1 (TIF 149 kb) [file 12012_2025_9989_MOESM1_ESM.tif]
